# Analysis of COVID-19 Vaccination Status Among Parents of Hospitalized Children Younger Than 5 Years With SARS-CoV-2 Infection During the Delta and Omicron Waves

**DOI:** 10.1001/jamanetworkopen.2022.42295

**Published:** 2022-11-16

**Authors:** Florie Solignac, Naïm Ouldali, Camille Aupiais, Paul Casha, Robert Cohen, Corinne Levy, François Angoulvant

**Affiliations:** 1 Pediatric Emergency Unit, Hôpital Universitaire Robert-Debré, Université de Paris-Cité, France; 2 Department of General Pediatrics, Pediatric Infectious Disease and Internal Medicine, Hôpital Universitaire Robert-Debré, Université De Paris-Cité, France; 3 Department of Pediatric Infectious Diseases, Sainte Justine University Hospital, University of Montréal, Québec, Canada; 4 Institut national de la santé et de la recherche médicale, Unité Mixte de Recherche 1123 Epidémiologie Clinique et Évaluation Économique Appliquées aux Populations Vulnérables, Université de Paris-Cité, France; 5 Department of General Pediatrics, Hôpital Universitaire Jean-Verdier, Université Sorbonne Paris Nord, Bondy, France; 6 Department of General Pediatrics, Hôpital Sainte Musse, Toulon, France; 7 Association Clinique et Thérapeutique Infantile du Val-de-Marne, Créteil, France; 8 Centre Hospitalier Intercommunal, Research Centre, Université Paris Est, Institut Mondor de Recherche Biomédicale-Groupe de Recherche Clinique-Groupe d’Etude des Maladies Infectieuses Néonatales et Infantiles, Créteil, France; 9 Groupe de Pathologie Infectieuse Pédiatrique, Paris, France; 10 Service of Pediatrics, Department Woman-Mother-Child, Lausanne University Hospital (Centre hospitalier universitaire vaudois), Switzerland; 11 Institut national de la santé et de la recherche médicale, Centre de Recherche des Cordeliers, Sorbonne Université, Université Paris Cité, France; 12 HeKA, Inria Paris, France

## Abstract

This cohort study uses COVID-19 Pediatric Observatory study data to analyze the vaccination status of parents with young children hospitalized for SARS-CoV-2 variants Delta and Omicron.

## Introduction

The appearance of the B.1.1.529 (Omicron) variant of SARS-CoV-2 in late December 2021 in France was associated with a rapidly increasing hospitalization rate among infants and children aged 0 to 5 years,^1^ for whom COVID-19 vaccines were not licensed. The association between parents’ vaccination status and severe infection in young children remains unclear. The aim of this study was to analyze COVID-19 vaccination status among parents of hospitalized children younger than 5 years with SARS-CoV-2 infection during the Delta and Omicron waves.

## Methods

This cohort study analyzed data from the COVID-19 Pediatric Observatory (PANDOR) study, a French national prospective surveillance study of hospitalized children with SARS-CoV-2 infection.^2^ All children younger than 5 years admitted between May 12, 2021, and February 14, 2022, with known information about parents’ COVID-19 vaccination status were included. The study was approved by the Institut national de la santé et de la recherche médicale ethics committee. All participants were provided written information, and oral consent was obtained. The study followed the STROBE reporting guideline.

To account for increasing numbers of parents vaccinated during the study period, we estimated hazard ratios (HRs) and 95% CIs for children who were hospitalized and had unvaccinated vs vaccinated parents by using a Cox proportional hazards regression model. The HRs are based on estimates of population prevalence of vaccination. From May 12, 2021, to February 14, 2022, the number of French adults aged 18 to 59 years who were vaccinated with 2 doses increased from 7% to 92%.^3^ Additional details are provided in the eMethods in the Supplement.

According to the spread of the different variants in France, we defined 2 periods: Delta (May 12 to December 12, 2021) and Omicron (December 20, 2021, to February 14, 2022). The week of December 13, 2021, was considered the washout period.

All statistical analyses were performed using Stata, version 16.1 (StataCorp LLC). A 2-sided *P* < .05 was considered significant.

## Results

During the PANDOR study period, 599 children were admitted; for 208, their parents’ COVID-19 vaccination status was known. Of 163 children younger than 5 years (78%), 63 were admitted during the Delta period, 6 during the washout period, and 94 during the Omicron period (Table 1). For the association of SARS-CoV-2 hospitalizations in children younger than 5 years with vaccinated vs unvaccinated parents, the HRs were 0.03 (95% CI, 0.02-0.06) during the Delta period and 0.21 (95% CI, 0.14-0.33) during the Omicron period (Table 2).

**Table 1.** Characteristics of Hospitalized Children Younger Than 5 Years With SARS-CoV-2 Infection During the Delta and Omicron Variant Periods

Characteristic	No. (%)	
	Delta period (n = 63)	Omicron period (n = 94)
Age, median (IQR), d	72 (33-329)	69 (33-306)
Age		
<90 d	36 (57)	56 (60)
90 d to 1 y	11 (18)	17 (15)
1-5 y	16 (25)	21 (25)
Sex		
Female	29 (46)	51 (54)
Male	33 (54)	41 (46)
Comorbidity	4 (6)	12 (13)
Hospital care^a^	40 (64)	35 (37)
Admission to a pediatric intensive care unit	10 (16)	8 (9)

^a^ Oxygen therapy, enteral nutrition, parenteral hydration, nebulization, or any combination.

**Table 2.** Hospitalization for SARS-CoV-2 Infection in Children Younger Than 5 Years by Parents’ Vaccination Status During the Delta and Omicron Variant Periods

Period	Children, No. (%)	HR (95% CI)^a^	*P* value
Delta			
Parents not vaccinated	51 (81)	1 [Reference]	NA
One or both parents vaccinated	12 (19)	0.03 (0.02-0.06)	<.001
Both parents vaccinated	8 (13)	NA	NA
One parent vaccinated	4 (6)	NA	NA
Omicron			
Parents not vaccinated	27 (29)	1 [Reference]	NA
One or both parents vaccinated	67 (71)	0.21 (0.14-0.33)	<.001
Both parents vaccinated	57 (60)	NA	NA
One parent vaccinated	10 (11)	NA	NA

Abbreviations: HR, hazard ratio; NA, not applicable.

^a^ Cox proportional hazards regression models were used to calculate HRs, with the number of adults aged 18 to 59 years vaccinated with 2 doses as the exposure and SARS-CoV-2 hospitalization in children younger than 5 years as the outcome. From May 12, 2021 (start of the generalized vaccination campaign for adults without age or comorbid conditions), to February 14, 2022, the percentage of French adults aged 18 to 59 years who were vaccinated with 2 doses ranged from 7% to 92%.^3^

## Discussion

During both the Delta and Omicron periods, parents’ vaccination status was associated with a reduced risk of hospital admission for SARS-CoV-2 in children younger than 5 years. A study performed before the Omicron wave showed an association of parent vaccination with a reduced risk of SARS-CoV-2 infection in children, with lower odds ratios likely due to older children in the sample.^4^ The association between parent vaccination and reduced risk of admission for SARS-CoV-2 in children younger than 5 years suggests that parents played a major role in transmitting SARS-CoV-2 to their young children during both waves,^5^ but the association between protection and vaccination seemed lower in the Omicron vs Delta period. The Omicron variant has been shown to be more transmissible, and the vaccine effectiveness against infection seems lower.^6^

This study has some limitations. It was a retrospective analysis of a prospective observational study. The existence of siblings; source of infection in the household; dates of parents’ vaccination; and existence of a booster, which is a bias considering the change of vaccine effectiveness over time and across variants, were not recorded. Furthermore, environmental changes, lockdowns, and compliance with social distancing according to parent vaccination status were not evaluated. These results should not be extrapolated to other variants, such as the predominant variants BA.4 and BA.5. Nonetheless, these results reinforce recommendations for widespread vaccination of parents of young children.

## Notes

Supplement.
eMethods
**eAppendix.** List of Investigators

## References

[1] Belay ED, Godfred-Cato S. SARS-CoV-2 spread and hospitalisations in paediatric patients during the omicron surge. Lancet Child Adolesc Health. 2022;6(5):280-281. doi:10.1016/S2352-4642(22)00060-8 35189084 PMC8856665

[2] Ouldali N, Yang DD, Madhi F, ; Investigator Group of the PANDOR Study. Factors associated with severe SARS-CoV-2 infection. Pediatrics. 2021;147(3):e2020023432. doi:10.1542/peds.2020-023432 33323493

[3] Le tableau de bord de la vaccination. Ministère de la Santé et de la Prévention. Accessed July 2, 2022. https://solidarites-sante.gouv.fr/grands-dossiers/vaccin-covid-19/article/le-tableau-de-bord-de-la-vaccination

[4] Hayek S, Shaham G, Ben-Shlomo Y, . Indirect protection of children from SARS-CoV-2 infection through parental vaccination. Science. 2022;375(6585):1155-1159. doi:10.1126/science.abm3087 35084938 PMC9799368

[5] Silverberg SL, Zhang BY, Li SNJ, . Child transmission of SARS-CoV-2: a systematic review and meta-analysis. BMC Pediatr. 2022;22(1):172. doi:10.1186/s12887-022-03175-8 35365104 PMC8975734

[6] Tseng HF, Ackerson BK, Luo Y, . Effectiveness of mRNA-1273 against SARS-CoV-2 Omicron and Delta variants. Nat Med. 2022;28(5):1063-1071. doi:10.1038/s41591-022-01753-y 35189624 PMC9117141

