# Supplementary material for: Analysis of COVID-19 Vaccination Status Among Parents of Hospitalized Children Younger Than 5 Years With SARS-CoV-2 Infection During the Delta and Omicron Waves
Source: JAMA Netw Open. 2022 Nov 16;5(11):e2242295. doi: 10.1001/jamanetworkopen.2022.42295 (PMC9669815; doi:10.1001/jamanetworkopen.2022.42295)
Supplement: Supplement. — eMethods eAppendix. List of Investigators [file jamanetwopen-e2242295-s001.pdf]

Supplemental Online Content

Solignac F, Ouldali N, Aupiais C, et al. Analysis of COVID-19 vaccination status among parents of hospitalized children younger than 5 years with SARS-CoV-2 infection during the Delta and Omicron waves. *JAMA Netw Open*. 2022;5(11):e2242295. doi:10.1001/jamanetworkopen.2022.42295

eMethods

eAppendix. List of Investigators

This supplemental material has been provided by the authors to give readers additional information about their work.

© 2022 Solignac F et al. *JAMA Netw Open.*

eMethods

Data were obtained from the PANDOR observatory which is a French national prospective surveillance of children hospitalized with SARS-CoV-2 infection. This observatory includes all children hospitalized with a confirmed diagnosis of SARS-CoV-2 infection, defined by a positive real-time reverse transcriptase polymerase chain reaction (RT-PCR) result for SARS-CoV-2 on a nasopharyngeal swab or by pediatric patients diagnosed with MIS-C according to World Health Organization criteria. For this study, we included only pediatric patients younger than 5 years whose vaccine status of parents was known. For each patient, an electronic case report form was prospectively demographic characteristics, comorbidities, hospital care, admission to pediatric intensive care units (PICUs) and parents' vaccination status were recorded.

The study was approved by the Institut National de la Santé et de la Recherche Médicale ethics committee for evaluation, the institutional review board (IRB00003888), and was registered at ClinicalTrials.gov (NCT04336956). A written information form validated by the ethics committee was given to all participants. Oral consent was obtained from study participants; no family members or participants refused to participate.

Proportional hazards assumption has been tested using Schoenfeld Residuals method. Results showed no evidence that the proportional-hazards assumption has not been violated with p-value over 0.05.

Data will be available upon reasonable request to francois.angoulvant@chuv.ch

Main Stata code lines using Stata 16.1 (StataCorp)

- stset date [fw=number], failure(covidH)
- stcox vaccin
- estat phtest
- stphplot, by(vaccin)

© 2022 Solignac F et al. *JAMA Netw Open.*

eAppendix. List of Investigators

In addition to the authors, the following collaborators participated in the investigator group of the PANDOR study
Investigators: None of them received compensation for their role in the study : Raphaël Anxionnat, M.D., Brigitte Auburtin, M.D., Benjamin Azemar, M.D., Catherine Barrey, M.D., Abdelmalek Belgaid, M.D., Sakina Benkaddouss, M.D., Grégoire Benoist, M.D., Philippe Bensaid, M.D., Biot Blandine, M.D, Valérie Biran, Ph.D., M.D., Jeanne Bordet, M.D., Philippe Blanc, M.D., Cécile Bost-Bru, M.D., Gérald Boussicault, M.D., Camille Brehin, M.D., Ph.D., Jacques Brouard, M.D., Ph.D., Aymeric Cantais, M.D., Ph.D., Bernard Caurier, M.D., Adela Chahine, M.D., Lorelei Charbonnier, M.D., Claire Charreton, M.D., Claude Chenel, M.D., Didier Chognot, M.D., Laure Cohen, M.D., Dana Dabbagh, M.D., Yvan De Feraudy, M.D., Marie Delattre, M.D, Céline Delestrain, M.D., Ph.D., Emmanuelle Dessioux, M.D., Jéremy Do-Cao, M.D., Marie-Aliette Dommergues, M.D., Aurélie Donzeau, M.D., Anne Drouard, M.D., François Dubos, M.D., Ph.D., Charlène Dupré, M.D., Xavier Durrmeyer, M.D., Ph.D., Florence Elmerich, M.D., Sophie Enchery, M.D., Sophie Fruscione, M.D., Florent Girard, M.D., François Gouraud, M.D., Richard Gruss, M.D., Margaux Guerder, M.D., Patricia Guglielmino, M.D., Véronique Hentgen, M.D., Ph.D., Valentine Hoeusler, M.D., Fréderic Huet, M.D., Ph.D., Charlotte Idier, M.D., Bérangère Jany, M.D., Etienne Javouhey, M.D., Ph.D., Nicolas Kalach, M.D., Rémi Kom, M.D., Ekaterina Kutsia, M.D., Anis Larakeb, M.D., Vanessa Latry, M.D., Camille Le Stradic, M.D., Aurélie Lemaire-Weber, M.D., Jean-Marie Lepage, M.D., Stéphane Leteurtre, M.D., Ph.D., Camille Loeile, M.D., Sylvie Louf, M.D., Zoha Maakaroun-Vermesse, M.D., Alexis Mandelcwajg, M.D., Mariana Marin, M.D., Cécilia Marques, M.D., Anne Martha Sandrine-, M.D., Martinat Laurence, M.D., Masserot Caroline, M.D., Laurence Mathivon, M.D., Pauline Meslin, M.D., Nathalie Mestre, M.D., Karen Milcent, M.D., Ph.D., Marie Mizzi-Rozier, M.D., Françoise Monceaux, M.D., Marie Monfort, M.D., Emmanuelle Mougenot, M.D., Eric Moulène, M.D., Florence Moulin, M.D., Elodie Nattes, M.D., Sophie Odorico, M.D., Marie-Clothilde Orcel, M.D., Medhi Oualha, M.D., Ph.D., Antoine Ouziel, M.D., Chantal Peigne, M.D., Béatrice Pellegrino, M.D., Isabelle Petit, M.D., Didier Pinquier, M.D., Charlotte Pons, M.D., Valérie Rabier, M.D., Blandine Robert, M.D., Christine Roche, M.D., Sylvie Roullaud, M.D., Danielle Roybet, M.D., Jonathan Rozental, M.D., Stéphane Sanchez, M.D., Jean-Marc Schneider, M.D., Bertrand Soto, M.D., Marie Szulc, M.D., Joelle Terzic, MD.D,

Caroline Thach, M.D., Léa Thevenet, M.D., Xavier Torterüe, M.D., Antoine Tran, M.D., Nicoleta Ursulescu, M.D., Cécile Valentin, M.D., Mathieu Verdan, M.D., Cindy Verney, M.D., Olivier Vignaud, M.D., Sébastien Walser, M.D., Mohamed-Amine Yangui, M.D., Brigitte Zimmermann, M.D., David Dawei Yang, M.D., Fouad Madhi, M.D., Michael Levy M.D.,Ph.D., Jean Gaschignard, M.D.,Ph.D., Irina Craiu, M.D., Tamazoust Guiddir, M.D., Cyril Schweitzer, M.D.,Ph.D., Arnaud Wiedemann, M.D., Ph.D., Mathie Lorrot, M.D., Ph.D., Anne-Sophie Romain, M.D., Aurélie Garraffo, M.D., Hervé Haas, M.D., Sébastien Rouget, M.D., Loïc de Pontual, M.D., Ph.D., Alain Martinot, M.D., Ph.D., Julie Toubiana, M.D., Ph.D., Laurent Dupic, M.D., Philippe Minodier, M.D., Manon Passard, M.D., Alexandre Belot, M.D., Ph.D., Camille Jung, M.D., Mayssa Sarakbi, M.D., Sarah Ducrocq, M.D., Nevena Dakova, M.D., Imen Jhaouat, M.D Nathalie Garrec, M.D., Elisabeth Caron, M.D., Vincent Gajdos, M.D., Ph.D.
